# Long Multi-Stage Training for a Motor-Impaired User in a BCI Competition

**DOI:** 10.3389/fnhum.2021.647908

**Published:** 2021-03-25

**Authors:** Federica Turi, Maureen Clerc, Théodore Papadopoulo

**Affiliations:** Université Côte d'Azur, Inria, France

**Keywords:** brain-computer interface, mental imagery, MI-BCI, event-related desynchronization/synchronization (ERD/ERS), long training, impaired subject, BCI competition, cybathlon

## Abstract

In a Mental Imagery Brain-Computer Interface the user has to perform a specific mental task that generates electroencephalography (EEG) components, which can be translated in commands to control a BCI system. The development of a high-performance MI-BCI requires a long training, lasting several weeks or months, in order to improve the ability of the user to manage his/her mental tasks. This works aims to present the design of a MI-BCI combining mental imaginary and cognitive tasks for a severely motor impaired user, involved in the BCI race of the Cybathlon event, a competition of people with severe motor disability. In the BCI-race, the user becomes a pilot in a virtual race game against up to three other pilots, in which each pilot has to control his/her virtual car by his/her mental tasks. We present all the procedures followed to realize an effective MI-BCI, from the user's first contact with a BCI technology to actually controlling a video-game through her EEG. We defined a multi-stage user-centered training protocol in order to successfully control a BCI, even in a stressful situation, such as that of a competition. We put a specific focus on the human aspects that influenced the long training phase of the system and the participation to the competition.

## 1. Introduction

Mental-Imagery based Brain-Computer Interfaces (MI-BCIs) control an external device by specific EEG components generated by mental imagery tasks performed by the user (Pfurtscheller and Neuper, 2001). Sensorimotor rhythms (SMRs) modulate the power of the ongoing EEG signal over sensorimotor areas (i.e., mu-rhythm and beta-rhythm) (Yuan and He, 2014). They occur during mental imagery tasks, such as mental arithmetic or mental rotation (Faradji et al., 2009) and motor imagery (Neuper et al., 2006) (Wang et al., 2020). This modulation of power in given frequency bands and spatial locations can be used to identify the mental task that caused this change in the brain rhythms. The power decrease is called an event-related desynchronization (ERD), while a power increase is called event-related synchronization (ERS) (Pfurtscheller and Da Silva, 1999).

There are many applications which use MI-BCIs, such as neurorehabilitation (Van Dokkum et al., 2015), control of external devices (Cincotti et al., 2008), virtual reality (Leeb et al., 2007), and gaming (Kauhanen et al., 2007). However, there are some limitations affecting the diffusion of such systems in real life setups. Among them there is the high intra- and inter-subject variability, preventing their common use in daily life (Saha and Baumert, 2019). The experimental setting, the psychological state and neurophysiological parameters all have an influence on the SMRs, which thus vary over time and across subjects, affecting the performance of MI-BCI systems. Another important parameter impacted by this variability is the design of a MI-BCI for an impaired subject. The system necessarily requires a definition phase in order to find the tasks most adapted to the subject, considering his/her neurological response but also his/her possibility to carry out specific tasks and then a training is fundamental to use the SMR-based BCI system. Moreover, to further improve the skill of modulating sensorimotor rhythms (Wolpaw and Wolpaw, 2012), a substantial training is required. Nevertheless, the basic mechanism of SMR learning is not clear. Many studies investigated on the motor learning process that promote plasticity in the sensorimotor networks and improve both motor and perceptual skills (Ostry and Gribble, 2016) proving that BCI skill acquisition effectively allows to improve the BCI performance also in impaired subjects. Yet, subject-specific training sessions may be required because the induction of plasticity varies significantly across subjects (Saha and Baumert, 2019).

Our work was focused on the design of a MI-BCI combining mental imagery and cognitive tasks for a severely motor impaired user, in preparation for the 2*nd* edition of the Cybathlon BCI race event, during a practice competition, the BCI Series, which took place as a satellite event preceding the BCI Graz conference in September 2019. The Cybathlon (Riener, 2016) is an international competition for people with severe motor disability who, equipped with assistive technology, compete in different events, such as the BCI race. In the BCI race, the user of the system becomes a pilot in a virtual race against up to three other pilots, in which each pilot controls his/her virtual car by his/her mental tasks. The virtual car is controlled on the race track through four different commands (go straight, turn right, turn left, and switch on the lights). By default, the car moves at constant speed on the track. A wrong control command sent by the BCI system is sanctioned by a reduction in speed, making the vehicle proceed slower on the track.

In this paper, we present the sequence of procedures we followed to realize an effective MI-BCI, from the selection of the pilot to the actual control of the video-game in the BCI Cybathlon series, with a particular focus on the long training phase. We defined a multi-stage user-centered training protocol in order to successfully control a BCI, even in a stressful situation, such as that of a competition.

## 2. Pilot Selection

Pilot selection started by asking Dr. Mariane Bruno of the Pasteur University Hospital in Nice, France, to present to us some of her patients with the disabilities listed by the Cybathlon competition, who would be both motivated and physically able to sustain the competition and its constraints (a long training, plus traveling to the competition site). Three motor-impaired women entered in this selection process. The selection process itself consisted in a few sessions of the Graz BCI protocol (Pfurtscheller and Neuper, 2001) as implemented in OpenViBE (Renard et al., 2010). This protocol tests the ability of the subject to achieve left and right hand motor imagery. The data was collected at the hospital in two half-day sessions and the signals were further analyzed offline using time-frequency plots to check visually that there was some signal to discriminate between the tasks.

One subject was excluded due to high spastic muscular activity, which generated too much artifactual EMG signal. Based on those data, two subjects were contacted to go further and have more training sessions, but one of them finally withdrew, because training for the competition appeared too strenuous. All further training was done with the only remaining subject, our pilot.

Our pilot is a 32 year-old woman, with limb girdle muscular dystrophy since the age of 7. She has no cognitive disability but severe motor disabilities. She does not control the movement of arms and feet, and can only perform a clamping movement with her hands, very limited on the left one. She controls her electric wheelchair with the index finger of the right hand, but she needs to be assisted for all daily activities, as for instance eating and drinking. Nonetheless she has no language disability and she has a strong personality. Moreover, she participated to different sports competitions for disabled people but did not have any experience with BCI. She has quite a competitive spirit, which is important to keep the motivation and sustain the long training sessions that we organized in the 3 months before the Graz Cybathlon event.

## 3. Training Protocol

To efficiently train our pilot, we deployed a multi-stage training strategy, that consisted in an investigation phase to determine the subject-dependent specific mental and cognitive tasks, followed by a training phase using those specific tasks.

### 3.1. Investigation Phase

The investigation phase is fundamental to define the most suitable MI tasks for the subject. Indeed, the mental tasks must fulfill three criteria: the subject must be able to perform each task and be comfortable with it, the individual mental task must produce a recognizable brain pattern and it must not cause undesirable side effects, like spasms, discomfort or stress (Schwarz et al., 2016).

We collected data over several sessions in 1 month, from the middle of June to the middle of July 2019. This phase took time because this experience was new both for the user, who had never used a BCI system before, and for our team. Indeed, it was the first time we worked with a disabled person, which obviously requires specific attention. Therefore, a preliminary phase was necessary to create collaborative relationship between the team and the user, to allow the user to become more familiar with the hardware and also to allow the team to understand how to effectively manage this type of experience, defining a suitable experimental protocol (Lotte et al., 2013; Schwarz et al., 2016; Perdikis et al., 2018).

The experiments took place in a room located in the pilot's living center “Centre René Labreuille” in Le Cannet, France. During each session, the EEG signal of the subject was recorded from a ANT-Waveguard cap with a Refa8 amplifier (512 Hz sampling rate). To lower the impedance between the electrodes and the subject's skin below 10 kΩ, a conductive gel was applied to the ground (FPz) and to the 13 electrodes placed in positions F7, Fz, F3, F4, F8, T7, C3, Cz, C4, T8, P3, P4, Pz. Two EMG electrodes were placed on the user's hands to check for the presence of involuntary movements.

The following MI tasks have been tested and already used in Schwarz et al. (2016) and Friedrich et al. (2013):

- MI of right hand (RH): close and open right hand, simulating the clamping movement.
- MI of left hand (LH): close and open left hand, simulating the clamping movement.
- Language (LAN): imagination of words that begin with a specific letter.
- Auditory (MUS): imagining singing a song.
- MI of both feet: move both feet.
- Calculus: imagination of incrementally summing numbers.
- No control (NC): relax.

**Figure 1:**
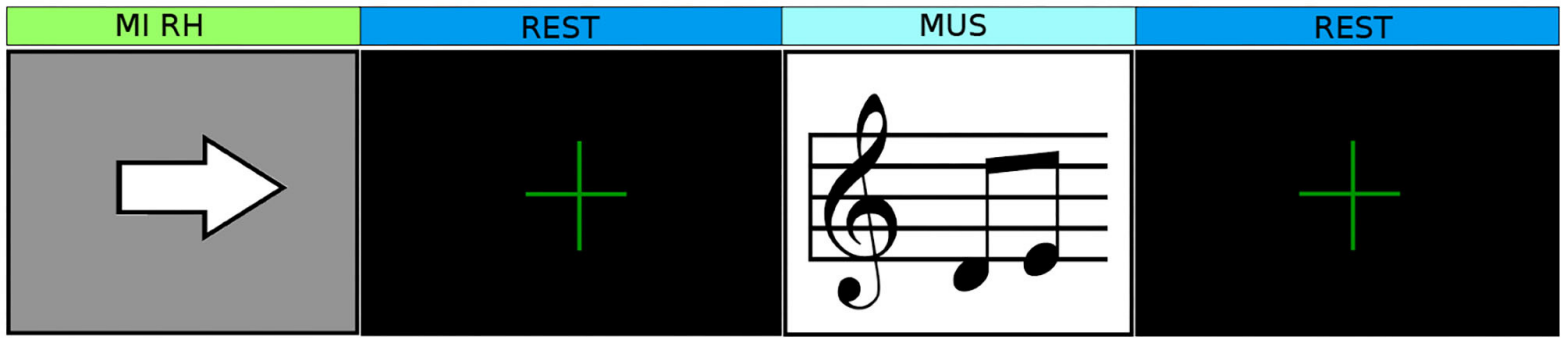
Example of an experimental paradigm applied in the investigation phase. We tested different time intervals of 5 s of tasks and 10 s of rest, 3 s/5 s, 3 s/10 s in order to determine the time interval that elicited prominent brain patterns.

The tasks were combined in different experimental paradigms, that were tried, in random order, during the first three sessions (S01, S02, and S03). The subject had to perform the mental tasks following the experimental paradigm that generally consisted in the combination of one or two control tasks interleaved by a no control task. In the no control task (NC), the user was asked not to engage in any MI task, but to achieve a relaxed state while gazing at a fixation cross on the screen. An example of an investigation paradigm is detailed in Figure 1. We tried different intervals between tasks in order to identify the interval combination that created more prominent brain responses. The paradigm was repeated 10 times in each run. Then, to identify the brain pattern of each task, we computed time-frequency plots to perform the event-related (de)synchronization (ERD/ERS) analysis. From empirical observations, the MI tasks of each investigation paradigm that did not produce an distinguishable (de)synchronization (ERD/ERS) activation on the EEG were considered not suitable for the user, and only MI tasks that produced a distinguishable pattern have been selected.

To create an efficient and adaptive BCI system, the selection criteria of the four tasks are: being the most distinguishable on the EEG and the easiest to realize for our pilot. For instance, some tasks such as the calculus created a lot of stress for the subject, the feet and left hand MI were also really complicated for our subject and were consequently considered as unsuitable tasks at this stage.

**Figure 2:**
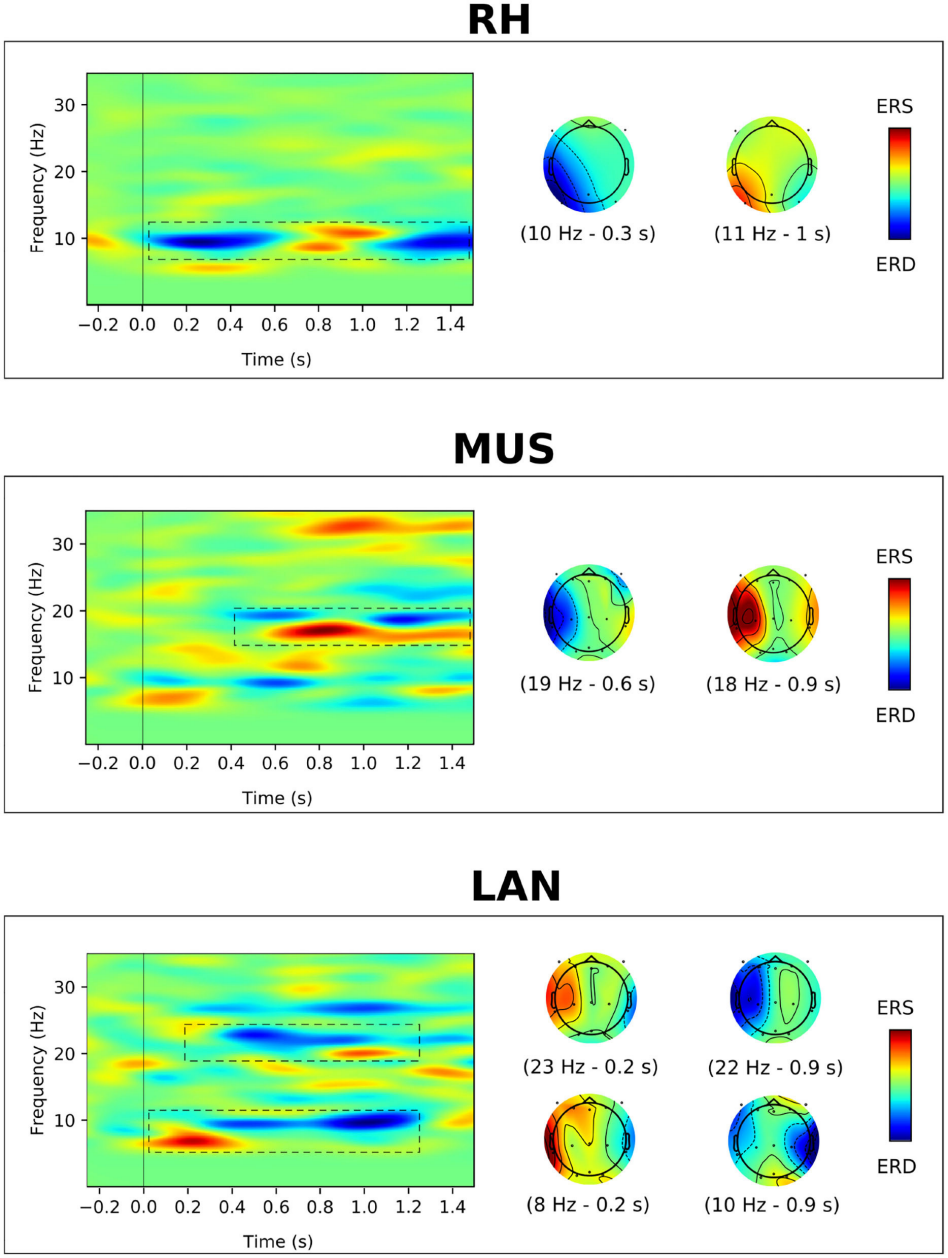
Average ERD/ERS maps calculated for MI of right hand (RH), auditory imagination (MUS), and word association (LAN). For each task, the pattern of activation is recognizable by dashed boxes in the frequency-time plot and the scalp topographies indicate the distributions of ERD/ERS at specific times and frequencies.

Finally, at the end of this investigation phase, the mental tasks suitable for our pilot were RH, MUS, LAN. These tasks provided a specific brain pattern in the pilot's EEG, as it can be seen on the ERD/ERS maps in Figure 2, and the subject was comfortable performing them. In addition these three MI tasks, NC task was considered as the fourth task suitable for our pilot.

### 3.2. Training Phase

The objective of the training phase was to train the subject to perform the mental tasks selected in the investigation phase. In this phase, the pilot had to perform many MI tasks without any feedback, aiming both at improving her ability to manage the tasks and at creating the training set to calibrate the BCI classifier.

**Figure 3:**
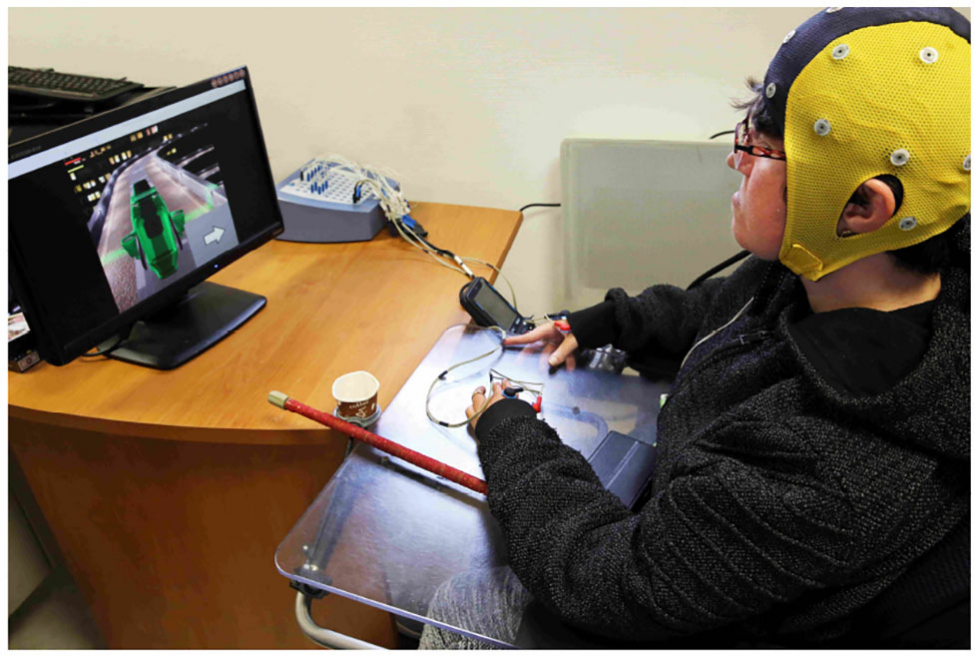
Experimental setup of the MI-BCI system. The pilot is wearing the EEG cap and EMG electrodes are placed on her hands.

The data were collected with the same hardware described in the previous investigation phase (see Figure 3). The sessions took place once a week from the middle of June to the end of August 2019 for a total of eight sessions.

Each training session lasted at least 2 h and 30 min, considering a break interval of around 10 min between each run, the time to set up the cap on the subject before the data acquisition and also the time to remove the cap and the gel after each session. Our pilot was really motivated at each session, but at the end of the session she was usually tired, in particular for sessions happening in the morning and during a very hot period. During some sessions the subject reported lack of concentration due to external noise, fatigue or hot temperature, in this case it was fundamental to take a break and to recover a more comfortable ambiance with all the team, improving her motivation and reducing her state of fatigue.

At the beginning (sessions S04 and S05), the experimental protocol consisted in 5 runs with the combination of 4 commands, but the subject reported that it was hard because it required a lot of concentration. Therefore, from sessions S06 to S07, the protocol consisted in 4 runs (RH-NC, RH-MUS-NC, RH-LAN-NC, and RH-MUS-LAN-NC) and we collected 10 trials per task and run.

**Figure 4:**
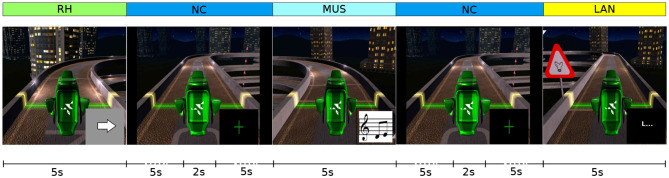
Experimental 4-class paradigm applied in the training phase. The user had to perform each control task (RH, MUS, LAN) for 5 s. Each task was associated to an image made by combining the task icon with an image extracted from the game at the proper time instant. The rest interval, corresponded to the no control task (NC), has a total duration of 12 s. After 5 s of this rest interval, a green cross appeared on the screen for 2 s to improve the concentration of the pilot.

In the last sessions, we tried to reintroduce the LH motor imagery task. Indeed, the subject at this moment improved her control on the RH task and we wanted to test whether or not the control of LH task would also have improved. Hence, from sessions S08 to S13, the protocol consisted in 5 consecutive runs (RH-MUS-NC, RH-LA-NC, RH-MUS-LA-NC, RH-LH-NC, and RH-LH-LAN-NC). The objective was to find the 4-class combination with the highest performance. An illustration of the 4-class experimental paradigm is exemplified in Figure 4. The total duration of the 4-class experimental paradigm is around 39 s, in which the user had to perform each control task for 5 s, where the control task is represented by a small icon (an arrow pointing to the right, a music score,...) superimposed on images extracted from the game. These images were selected to show the moment at which the subject would have to perform the task, in order to get the subject accustomed to perform the right task at the right moment. The rest interval, corresponding to the no control task (NC), has a total duration of 12 s. After 5 s of this rest interval, a green cross appeared on the screen for 2 s.

In order to detect the ERD and ERS in the EEG associated to the individual mental tasks, the EEG signal was bandpass filtered with a Butterwoth bandpass filter of 4th order in six different frequency bands (8–12, 16–20, 20–24, 28–32, 32–36, 36–40 Hz). The ERD/ERS appear around 0.5 s after the beginning or the end of the mental task and last between 1.5 and 3 s. Therefore, we considered epochs of 2.5 s from the mental imagery onset, with steps of 0.5 s, in order to build a BCI system that reacts as fast as possible to the pilot's intent during the online game. The size of the window was empirically selected, observing the time-frequencies plot of the preprocessing data. It is important to underline that it is not possible to define a priori the size suitable for all the users, because it is a user-related parameter. A feature vector was constructed by computing the average power in each frequency band in two successive windows, in order to capture both ERD and ERS events. This feature vector was provided to a LDA classifier to classify the different tasks, for each task 400 samples have been considered. The LDA classifier was trained using 70% of the band-power features as training set, the remaining 30% data were used as a validation set.

A multi-class confusion matrix was computed to assess the performance reached by each task for the different experimental paradigms. In particular, to analyze the performance of the individual tasks per session, we considered the F-score (Equation 1), that is a statistical measure to evaluate the test's accuracy considering both the precision and the recall. *Precision* is the number of True Positives (TP) divided by all positive predictions (TP+FP) returned by the classifier, and *recall* is the number of True Positives (TP) divided by the number of all samples that should have been identified as positive (TP+FN). F-score is more suitable for multi-class problems than the overall accuracy because it is not dependent on True Negatives (TN), that can overestimate the performance of the system (Sokolova and Lapalme, 2009).

(1) $$\begin{array}{l} F\text{-}score=2\cdot \frac{precision\cdot recall}{precision+recall} \end{array}$$

**Figure 5:**
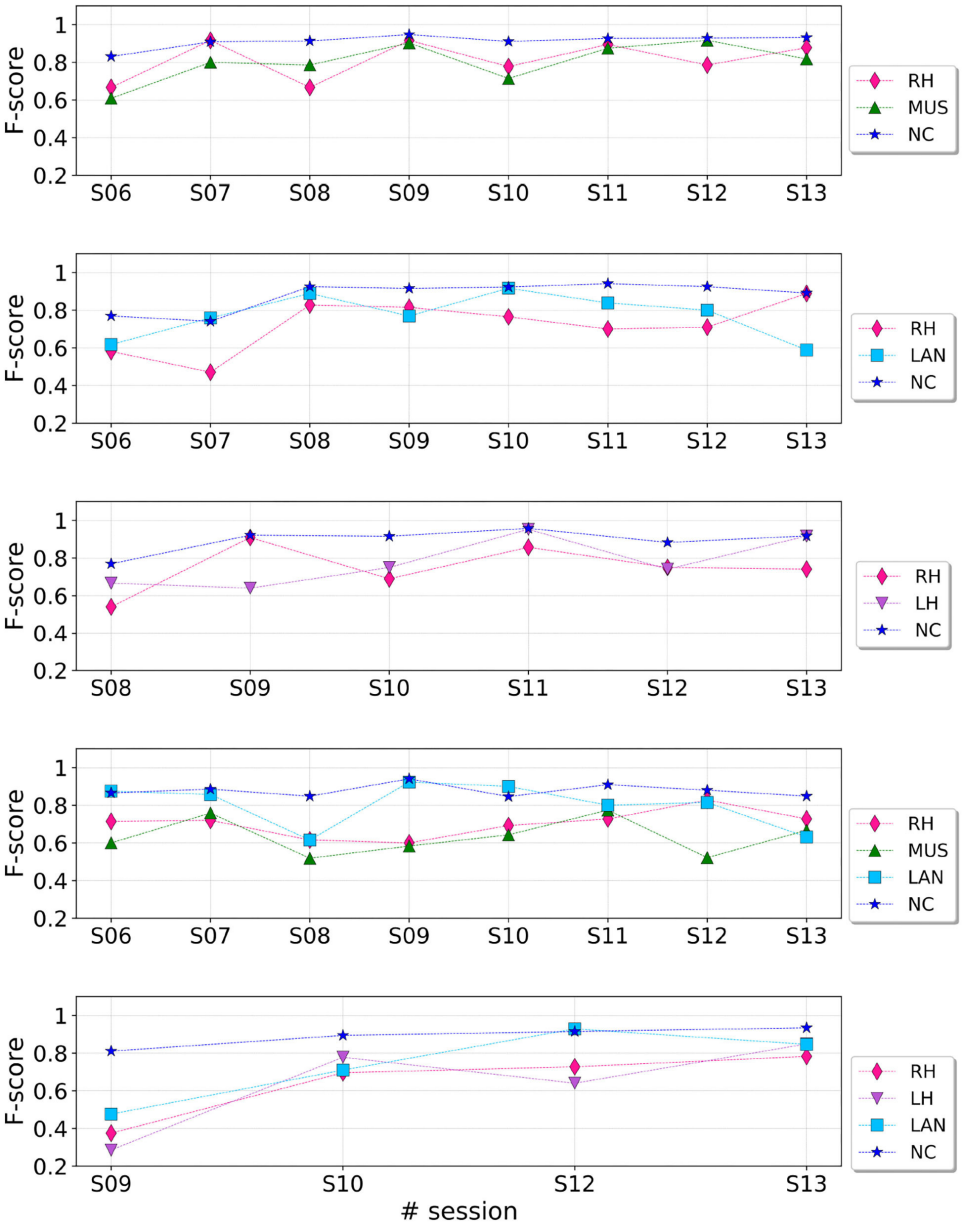
F-score values reached by each training paradigm across sessions.

Figure 5 shows the F-score achieved by the individual tasks across sessions. As a general remark, it can be noticed that, independently of the experimental paradigm, the user could better manage the NC task than the RH, MUS and LAN ones. Indeed, the F-score of the NC task (across all sessions and paradigms) was always above 0.8. This is a nice property as straight lines, which were associated to NC, tend to dominate in the race circuits.

We can furthermore notice that the performance reached in runs with three classes is generally more stable than the one obtained with four classes. Indeed, if we consider the 3-class paradigm RH-MUS-NC, the subject was able to manage all the three tasks across sessions. On the contrary, if we consider the 4-class paradigm RH-MUS-LAN-NC, the user managed better the NC and LAN tasks than the RH and MUS. This trend perfectly reflects the difficulty of the subject to perform runs with four tasks, as she declared. This is the reason why we designed the progressive training protocol detailed previously, in order to gradually manage 4-task control without requiring too much concentration and effort.

This strategy allowed the pilot to improve the classification performance for the 4-class combination RH-MUS-LAN-NC. Indeed, as shown in the fourth plot of Figure 5, we can notice an average improvement of performance from sessions S08 to S11 for the tasks RH and MUS, that the subject managed with difficulty at the beginning of the training.

**Figure 6:**
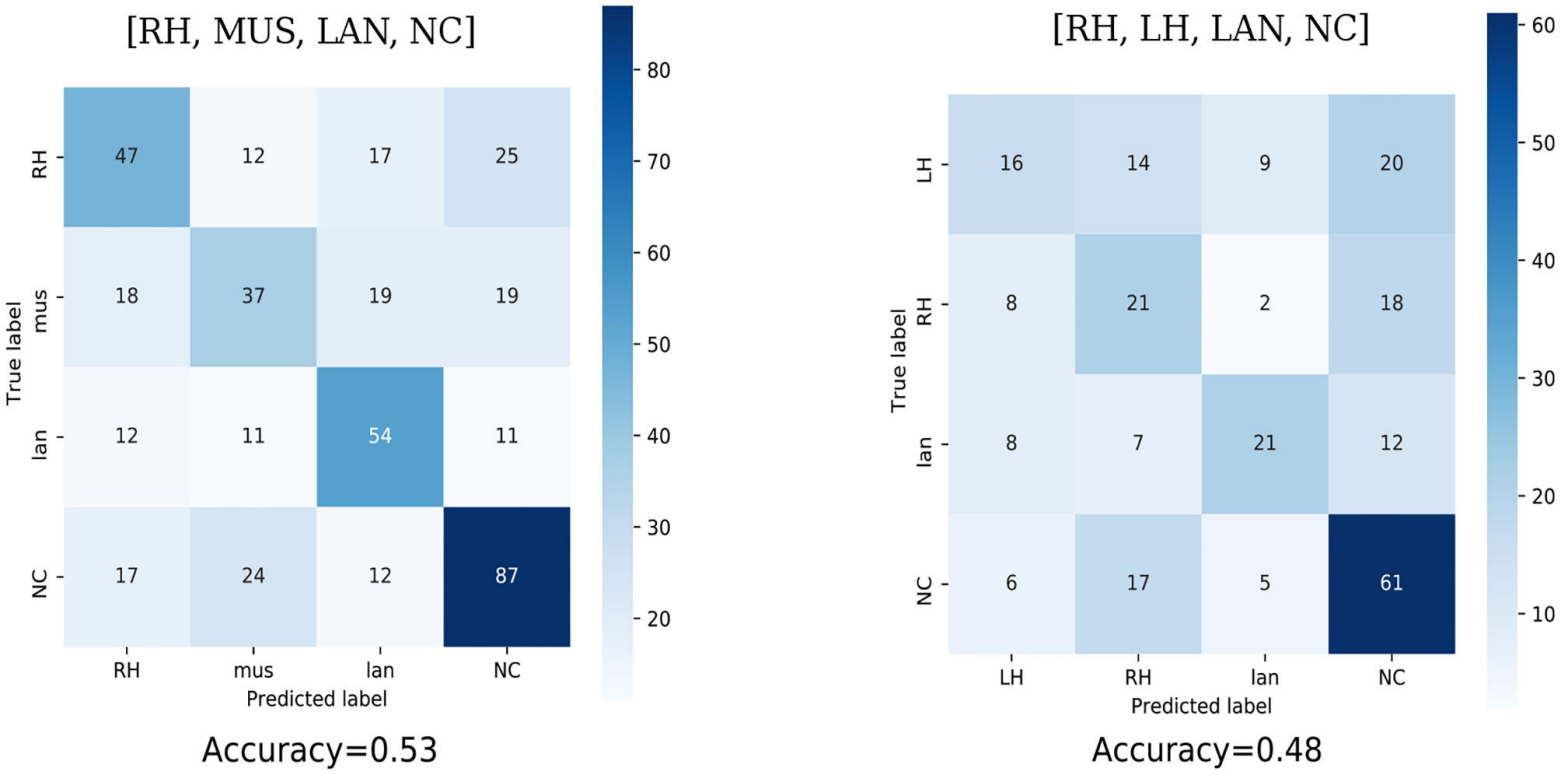
Confusion matrix of the two 4-class paradigms tested during the training phase, from sessions S08 to S13. Each confusion matrix reports the absolute values (numbers in black) and relative percentages (color scale) to evaluate the performance of the LDA classifier. All values on the diagonal represent the correctly classified trials. At the bottom the overall classification accuracy is given.

Finally, to evaluate the 4-class combination, the confusion matrix across sessions S08 to S13 were computed (see Figure 6). The results reached with the RH-MUS-LAN-NC are clearly better as shown by the better contrasted diagonal. Indeed, the RH-LH-LAN-NC paradigm not only displays a poor detection of the LH task, but also seems to induce some disturbance in the RH-NC discrimination. Accuracies are also reported to compare the classification among the 4-class combinations. It is computed as the sum of the correctly identified classes (TP+TN) over the all the classified classes (TP+TN+FP+FN). In our four classes case, it is the sum of the diagonal terms of the confusion matrix divided by sum all its terms. The results show a difference of 5% between the two paradigms, with an accuracy value equal to 53% for RH-MUS-LAN-NC and 48% for RH-LH-LAN-NC.

Therefore, the RH-MUS-LAN-NC paradigm which reached the highest performance was selected as the paradigm to apply in our closed-loop gaming BCI. Finally, the user agreed on this choice because she declared to be much more comfortable with the RH-MUS-LAN-NC combination than with the RH-LH-LAN-NC one.

#### 3.2.1. Closed-Loop BCI Game

**Figure 7:**
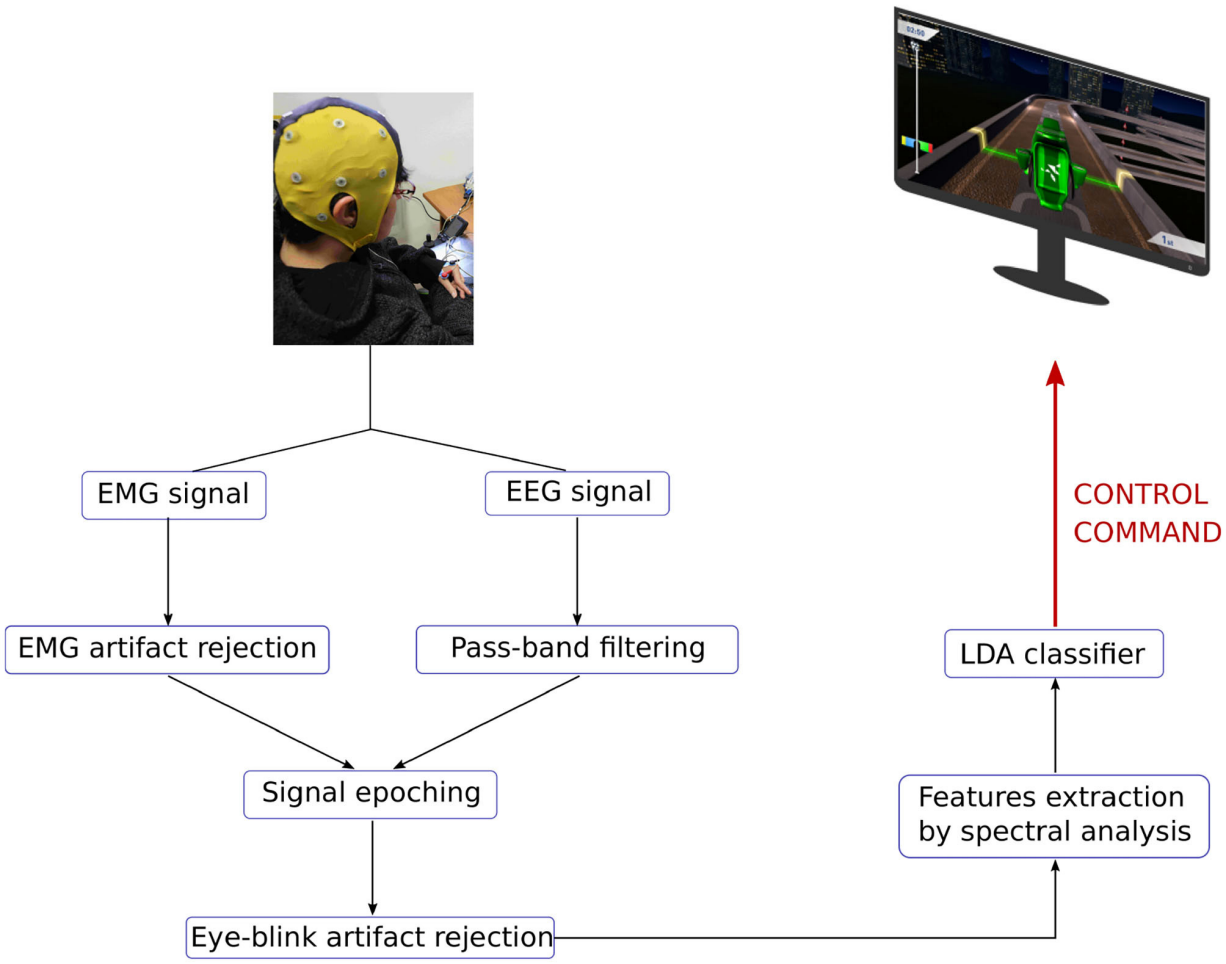
Outline of the closed-loop BCI system.

Figure 7 shows an illustration of the closed-loop BCI game. Basically, the EEG signal is acquired from the 13 channels (the same as in the training phase) and is bandpass filtered. In parallel, the EMG signal is processed in order to detect possible hand movement artifacts. Epochs corresponding to EMG artifacts are removed. Then, each retained epoch is tested for eye blink artifacts. EMG and EEG artifact rejection is detailed in the following paragraph. Each processed epoch provides a feature vector which is classified by the LDA classifier trained using the training dataset. Finally, the classification outputs are mapped to the video-game commands. In particular NC task was applied to move the car along the straight portions of the race track, RH task to turn the car right, MUS task to turn the car left and LAN task to switch on the lights.

Software difficulties were encountered in our initial implementation which made the BCI system unstable after a few minutes because of an excessive memory consumption which induced unsustainable latencies. As these difficulties arose only with the Windows operating system, we decided to run the BCI system on a Linux OS instead. But as the EEG acquisition software required a driver only available on Windows OS, we had to rely on two computers keeping a Windows computer to acquire the EEG and EMG signals. The two computers were linked using a TCP/IP connection. This required some network hardware and configuration, which added significant complexity to our system (and brought additional stress to our team during the live event in Graz). In the end, everything worked as expected, but much time which could have been better devoted to training the pilot *in situ* was lost. We learned the lesson that an effective BCI system must also be simple to setup, and are now working toward that goal.

**Figure 8:**
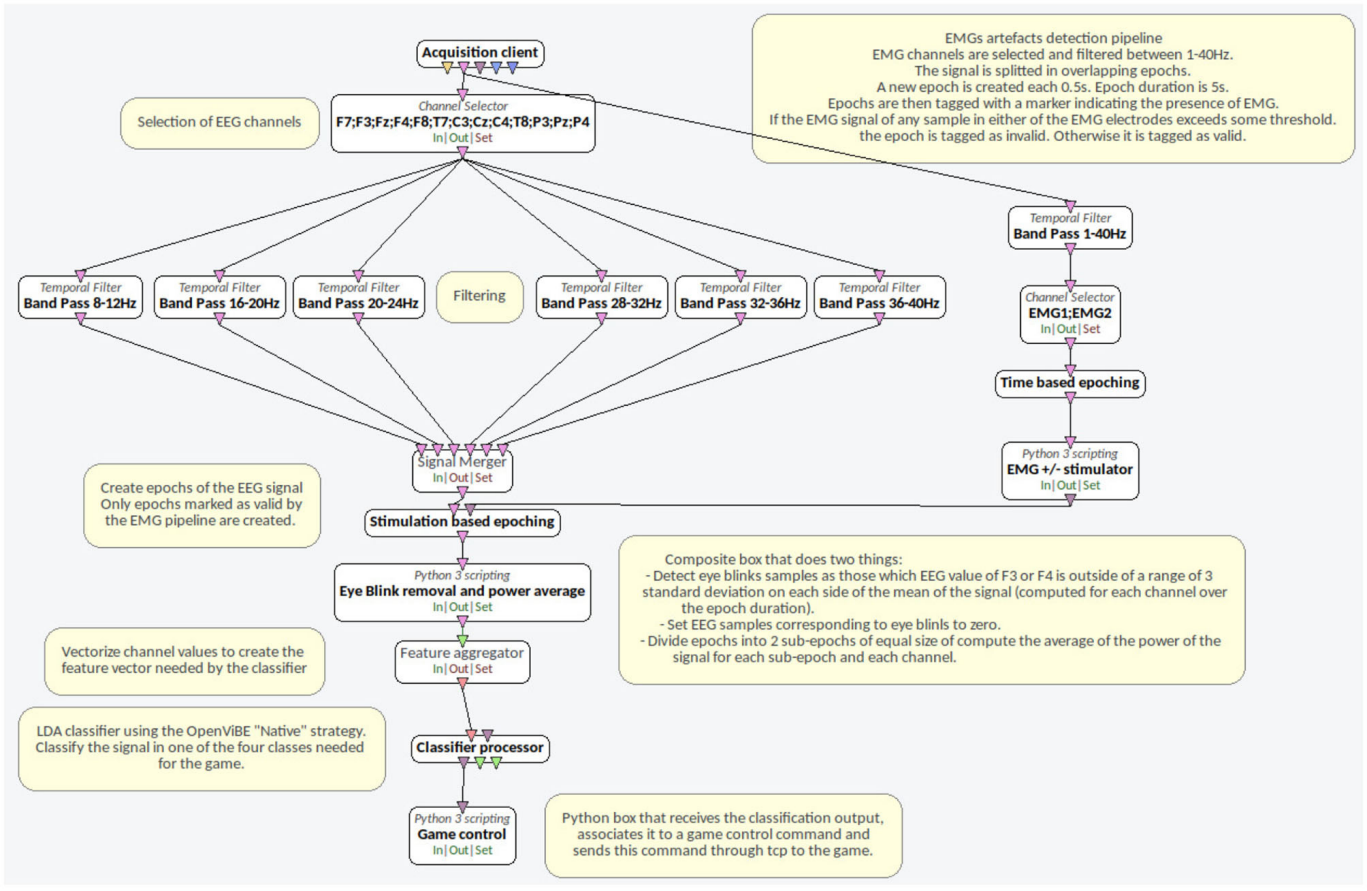
Openvibe scenario developed to control the BCI system.

The OpenViBE (Renard et al., 2010) scenario developed to control the game is detailed in Figure 8.

#### 3.2.2. Artifact Rejection Framework

To follow the Cybathlon BCI race regulations, we deployed an artifact rejection framework into the BCI system that includes both electromyogram (EMG) artifact rejection and eye-blink artifact rejection. The artifact rejection subsystem detects eye-blinks and/or EMG artifacts on the signals and prevents the BCI system to send any control command to the pilot's virtual car for a predefined time interval.

For the EMG artifact rejection, two adhesive surface electrode pairs were placed on both pilot's hands between the thumb and index fingers. We adopted this configuration because the only motor tasks achievable by the user were to close and open both hands, as in a clamping movement. These motor tasks were the real movements corresponding respectively to the RH and LH tasks.

The subject did two acquisitions, in which she performed voluntary hand movements at regular intervals in order to define an EMG threshold *T*_*EMG*_. The EMG rejection algorithm was defined to reject epochs for which the average EMG signal amplitude exceeds by two standard deviations the threshold *T*_*EMG*_, Thus no command can be sent to the game during such epochs.

The objective of the eye-blink artifact rejection was to detect the eye blinking on the EEG signals. In order not to overload the pilot with sensors, the EOG artifact rejection subsystem detects the presence of eye blinks on the frontal EEG electrodes F3 and F4, close to the left and right eyes. Artifact rejection was performed processing the EEG signals in the 8–12 Hz frequency band, in which the eye blinks of our subject was most prominent. For each epoch, the means and standard deviations of the F3 and F4 electrodes were computed. Time samples corresponding to instants in which the amplitude of F3 (respectively F4) was not in the range of the mean plus or minus three standard deviations of F3 (respectively F4) were discarded from the computation of the power features.

### 3.3. Cybathlon BCI Series

The Cybathlon BCI series event took place in Graz in September 2019. This BCI race offered the opportunity to showcase our research and development and gave the pilot an experience of a competition, in preparation for the Cybathlon 2020 event. Six international teams participated to this event and all teams had previously participated to Cybathlon 2016, except for NITRO 1 and NITRO-2 (our team). The race followed exactly the same rules as the Cybathlon BCI race. The pilots were competing together at most four at a time.

The criterion for winning the game was to complete the track in the shortest possible time, not exceeding 4 min (in which case, the distance along the track was used to rank pilots). The competition consisted in two phases: qualifications and finals. Two qualification races of three pilots were organized: the four first pilots in the qualification ranking took part to the final race A, the last two to the final race B.

**Figure 9:**
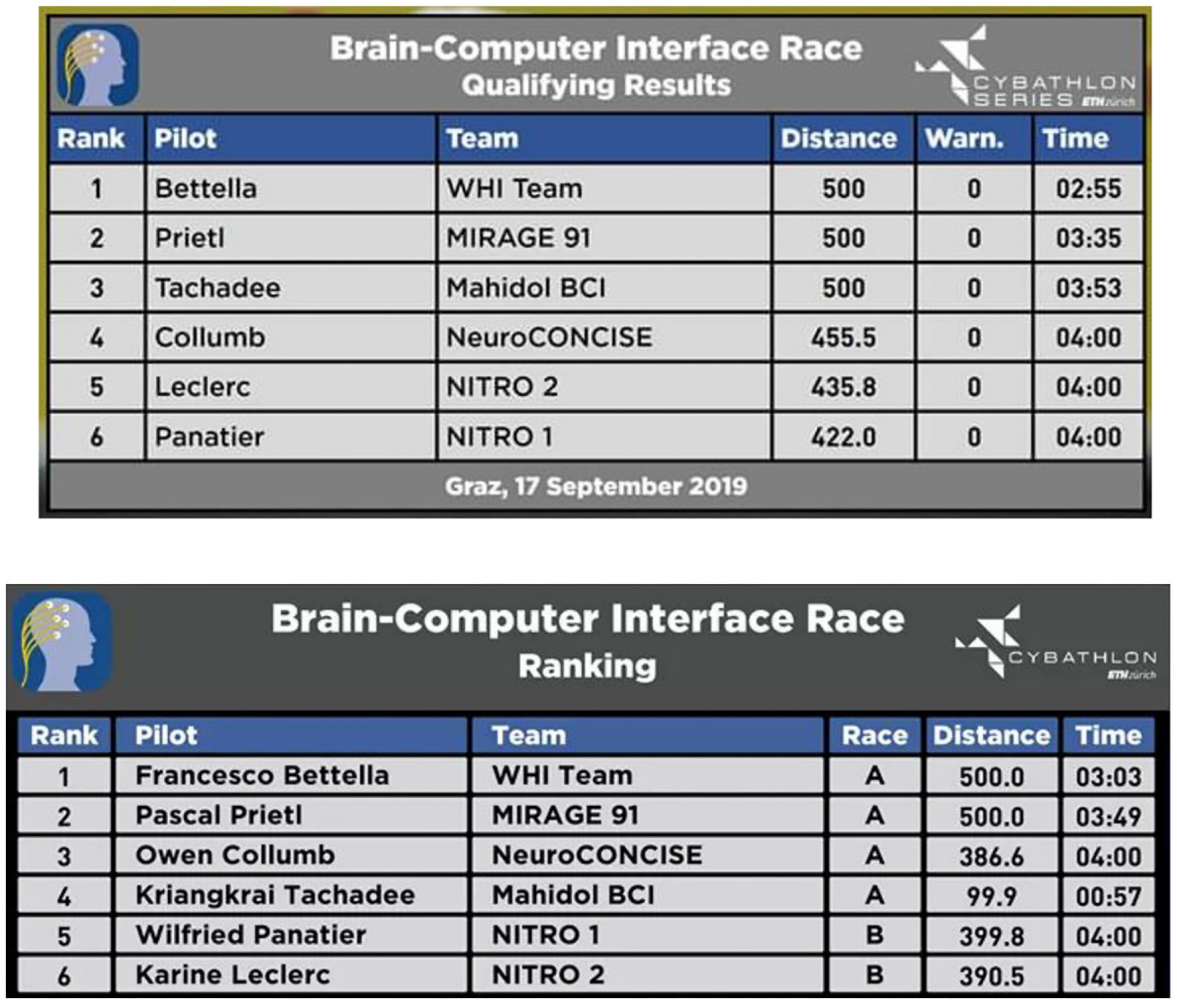
Cybathlon BCI series ranking.

The official results of the Cybathlon BCI series are shown in Figure 9. One pilot was disqualified during the final race. The pilots who reached the first and the second place finished the whole track with a very good timing. We reached the fifth position during the qualifier race and the last position during the final race.

One factor that prevented us from classifying in a higher position in the final race was the weakness of our hardware set up. Considering the channels configuration, in terms of limited number of electrodes of the EEG cap, we had a system with 13 channels, differently from all other teams that used 32 channels, except for one that used a 16 channels EEG cap. Many studies reported that the classification accuracy of a MI-BCI task can improve when increasing the number of channels (Meng et al., 2018). Moreover, differently from other teams, we had a complex setup of two computers connected through a network, that could have also impacted the classification accuracy. Moreover, we spent most of the test day trying to resolve network issues that arose in the context of the competition environment, so we had no time to train our pilot the day before the competition or to recalibrate the system, in fact we used a data set created combining all the training sessions to calibrate the system before the competition. Even on the day of the competition, passing the Tech-check (a test to see whether each team's system is able to communicate with the game infrastructure) proved to be difficult and was achieved at the last minute. On the positive side, once we ruled out the network problems, our system proved functional and stable during the whole race, contrary to some other teams which experienced some problems and had to repeat a qualification run to obtain their final result. Another success is that even if our pilot finished in last position, she led the race in both the qualification and final races till the last few seconds. Most probably, this was related to a concentration problem, as the race took place in a crowded amphitheater with a lot of cheering for the pilots, especially around the race end, and our pilot had not been trained in such an atmosphere. Indeed this can be considered the most important factor that impacted the performance during the race because, to efficiently control a MI-BCI system, a high level of concentration is required.

Nevertheless, the Graz experience was really useful for the future improvement of the system. We had the possibility to test our system in real life conditions, we understood the limits of our system and on what we need to work on to become more competitive for the Cybathlon race.

Finally, we also learned a lot on the human side of the race. Airplane travel, local accommodations, and land transport, while having been planned thoroughly and well in advance, were a source of stress for several pilots. We had to find solutions on the fly for several transportation or usual daily life issues.

### 3.4. Discussion and Perspectives

The long training phase and the BCI series in Graz provided us an enriching experience to understand the limits of our current BCI system. We identified several factors that influence the usability and the performance of our system and how these can be improved in the future.

We would like to underline that we had only 3 months to design the system, adapt it as best as possible to the pilot and train her, which is not a very long period for the preparation to this type of competition.

We did a long phase of training but could only train her to control the game itself for a few sessions (2 or 3) before the competition. For sure, learning to use the system and learning to “play” are two different tasks and therefore imply different levels of concentration. For instance, on the day of the competition, we noticed that during the last minutes, our pilot had more difficulties to stay concentrated. Concentration skills during the game could have been improved if we had more time to train the pilot with the game. The version of the game that was provided to teams at the time was not providing expected labels, thus data collected while using the game could not be used as training data. Consequently, pure game training time was limited.

There were many factors that influenced the stress condition of the pilot, impacting her ability to concentrate and consequently her performance. For instance, during both investigation and training phases, the acquisition took place in a standard room in a living center, as mentioned before. The room was not equipped for EEG experiments and not shielded for external sounds, consequently many times the training sessions were disturbed by external sounds that distracted the subject. Moreover, the whole training phase took place in summer and therefore in very hot and humid conditions, this condition decreased the pilot's concentration time-span mainly because of the inconvenience and discomfort of having to wear an EEG cap with gel during a heat wave.

Also, during the BCI series in Graz, many factors influenced her stress, such as competition stress, travel, and others logistic problems. In fact, this experience highlighted the problems faced by disabled people, particularly in terms of logistics (adapted transport and infrastructure) and special needs (lifts, wheelchairs, adapted taxis, and toilets, etc.). It also led us to realize the stakes of the organization of such an event.

Another factor that probably induced an increase of the overall stress of the pilot was the presence of many people and of noise during the competition. Indeed, the competition took place in an amphitheater room and each pilot was positioned in front of the public, and during the competition a person commented the race, whereas during all the training period, we tried to keep environmental disturbances as low as possible. Indeed, after the competition the pilot reported a state of stress and disappointment due to the fact that she had high difficulty to concentrate during the competition, due to the comments and cheering. This is justifiable since the pilot had to perform also cognitive tasks for which the concentration and calm are key elements. This aspect should be considered for future competition in order to put the subject in a comforting situation during the competition, so that each pilot can give the best performance.

In the BCI series in Graz, we noticed a great deal of variability between pilots. For example, residual motor abilities were highly variable from one pilot to another. Some pilots were able to fully use their arms, others could not move at all, some disabilities were congenital while others were recent. It is challenging to create a system that can be adapted to all situations. This confirms the importance of personalizing BCI systems, to tackle the needs of each user in any situation, such as a competition or real life.

The participation to this competition was a really exciting challenge and provided us a very informative experience in the development of a BCI for a disabled person. We understand that the role of the user is fundamental in a SMR-BCI system, confirming the need to develop user-centered systems in particular for disabled people that present different needs, based also on their disabilities.

After the BCI series, there were many aspects that we would have liked to improve in our system. On the human side, training the pilot to play the game with external disturbance (noise, and a cheering public) and improve her concentration capability would help her to maintain her maximum performance up till the end of the track. It would have also been productive to allow the pilots to train against each other in order to simulate real competitions. On the system side, we need to simplify our setup and remove the use of two computers linked by a network.

## 4. Conclusions

In this work, we deployed a MI-BCI system for a motor impaired user in the context of a BCI-game competition. A special focus was put on the long multi-stage training necessary to obtain an effective system. We presented and discussed our strategy to design an experimental user-centered experimental protocol. Moreover, we highlighted that the emotional state of the user, in terms of stress and concentration, directly impacts the performance of the system, in particular in a live competition.

## Data Availability Statement

The raw data supporting the conclusions of this article is available without undue reservation at https://project.inria.fr/cybathlonnitrodata. Note that the data is provided in raw form, and that a substantial amount of work is needed to process it and reproduce the research reported in this article. Please cite this article if you use the dataset in a research publication.

## Ethics Statement

The studies involving human participants were reviewed and approved by Comité Opérationnel d'Évaluation des Risques Légaux et Éthiques (COERLE) of INRIA. The patients/participants provided their written informed consent to participate in this study. Written informed consent was obtained from the individual(s) for the publication of any potentially identifiable images or data included in this article.

## Author Contributions

All authors listed have made a substantial, direct and intellectual contribution to the work, and approved it for publication.

## Conflict of Interest

The authors declare that the research was conducted in the absence of any commercial or financial relationships that could be construed as a potential conflict of interest.
